# Lessons learned from a pilot implementation of physical activity recommendations in axial spondyloarthritis exercise group therapy

**DOI:** 10.1186/s41927-021-00233-z

**Published:** 2022-01-17

**Authors:** Anne-Kathrin Rausch Osthoff, Theodora P. M. Vliet Vlieland, André Meichtry, Leti van Bodegom-Vos, Beatrice Topalidis, Stefan Büchi, Irina Nast, Adrian Ciurea, Karin Niedermann

**Affiliations:** 1 ZHAW, School of Health Sciences, Institute of Physiotherapy, Zurich University of Applied Sciences, Katharina-Sulzer-Platz 9, 8401 Winterthur, Switzerland; 2 Department of Orthopaedics, Rehabilitation and Physical Therapy, Leiden University Medical Center, Leiden, The Netherlands; 3 Department of Biomedical Data Sciences, Leiden University Medical Center, Leiden, The Netherlands; 4 Swiss Ankylosing Spondylitis Association, Zurich, Switzerland; 5 Clinic for Psychotherapy and Psychosomatics Hohenegg, Meilen, Switzerland; 6 Department of Rheumatology, Zurich University Hospital, 8091 Zurich, Switzerland

**Keywords:** Physical therapy, Ankylosing spondylitis, Assessment, Coaching, Counselling, Group exercise

## Abstract

**Background:**

The Ankylosing Spondylitis Association of Switzerland (SVMB) aimed to implement physical activity recommendations (PAR) within their exercise groups (EGs). The PAR promote exercise in all fitness dimensions at the correct dose. To implement the PAR within EGs, they were translated into a new EG concept with five key activities: (a) training for supervising physiotherapists (PTs), (b) correctly dosed exercises in all fitness dimensions, (c) exercise counselling, (d) bi-annual fitness assessments, and (e) individual exercise training, in addition to EG. All these activities were realized in close coordination with SVMB management.

**Objectives:**

To analyse the implementation success by evaluating adherence/fidelity, feasibility, and satisfaction at the patient, PTs, and organisational level.

**Methods:**

The five key activities of the new EG concept were developed, executed, and assessed after 6 months. The primary outcomes for implementation success were adherence of patients to the recommended exercise behaviour, self-reported by electronic diary; fidelity of PTs to the new concept, self-reported by diary; SVMB organisational changes. Secondary outcomes were feasibility and satisfaction with the new EG concept at all three levels. The tertiary outcome, to evaluate the effectiveness of PAR, was patient fitness, assessed through fitness assessments.

**Results:**

30 patients with axSpA (ten women, mean age 58 ± 9 years) and four PTs (three women, mean age 46 ± 9 years) participated. The patients' self-reporting of adherence to the PAR was insufficient (43%), possibly due to technical problems with the electronic dairy. The PTs' fidelity to the new EG concept was satisfactory. On all levels, the new concept was generally perceived as feasible and useful for supporting personalised exercise.The frequency of exercise counselling and the fitness assessments was found by patients and PTs to be too high and rigid. Patients' cardiorespiratory fitness [ES 1.21 (95%CI 0.59, 1.89)] and core strength [ES 0.61 (95%CI 0.18, 1.06)] improved over the 6 months.

**Conclusions:**

The pilot implementation of PAR showed acceptance and satisfaction to be sufficient, thus confirming the need for evidence-based EGs, provided by a patient organisation in order to support active PA behaviour. However, adaptations are necessary to increase its feasibility for nationwide implementation.

*Trial Registration*: SNCTP, SNCTP000002880. Registered 31 May 2018, https://www.kofam.ch/en/snctp-portal/search/0/study/42491.

**Supplementary Information:**

The online version contains supplementary material available at 10.1186/s41927-021-00233-z.

## Introduction

Patients with axial Spondyloarthritis (axSpA) have an increased risk of cardiovascular diseases [1]. In addition, evidence shows that axSpA has an impact on flexibility [2], balance [3], muscle strength [4], and cardio-respiratory capacity [5].

Exercise and effective drug treatment are recommended as the cornerstones of disease management [6, 7]. Indeed, the promotion of physical activity (PA) and specific exercise (cardiovascular, muscle strength, flexibility, and neuromotor exercise) should be an integral part of standard care throughout the course of disease [8]. Regarding cardiovascular exercise, evidence underpins that the appropriate intensity and frequency are relevant to reduce the risk of cardiovascular diseases in healthy people effectively; this might be valid for people with rheumatic and musculoskeletal diseases (RMDs) as well [9, 10]. Furthermore, evidence shows that exercise according to public health recommendations for health-enhancing PA is effective, safe, and feasible in people with RMDs [11]. Consequently, PA recommendations should be implemented, establishing a standard for PA and exercise promotion and education of health professionals for effectively delivering PA promotion and exercise across Europe.

The Ankylosing Spondylitis Association of Switzerland (Schweizerische Vereinigung Morbus Bechterew, SVMB) decided to implement the PA recommendations within their exercise groups. It was further decided to involve the SVMB members who currently do not participate in exercise groups at a later stage of time.

Previously, the groups, each consisting of between 5 and 25 patients with axSpA, met weekly to conduct 45–60 min of spinal flexibility and strength exercises, mostly land-based, few water-based, or combined, under the supervision of a physiotherapist (PT). Health insurers refunded the costs for the attendance of these exercise groups.

The overall aim of this implementation project was to establish the PA recommendations for patients with axSpA participating in exercise groups to perform exercises regularly and adequately dosed [12]. The PA recommendations were translated into a new SVMB exercise group concept, named "moveSVMB" (an acronym for *iMplementation Of physical actiVity rEcommendations in SVMB*). It incorporated the following key implementation activities: (a) 2 days of training for supervising PTs, (b) exercises in all fitness dimensions, according to the exercise guidelines [13], (c) quarterly individual exercise counselling with a supervising PT to support more individual exercise training, (d) bi-annual fitness-assessments, and (e) individual exercise training in addition to group exercise. All implementation activities were closely coordinatedby SVMB management and study staff.

It is well known that the implementation of innovations in clinical routine is a great challenge [14, 15], requiring a planned and systematic approach with clear strategies. Knowledge does not necessarily lead to changed behaviour [16–18]. In this project, the implementation process was determined following the cyclical approach of Grol and Wensing' implementation of change model [19] and included the following steps (and publications): (A) analysis of the current clinical practice and settings (as briefly outlined above, [20]); (B) exploration of facilitators and barriers towards PA and exercise from the stakeholders' perspective, i.e. patients with axSpA, PTs and rheumatologists [21], including the patients' perspective on technology-based exercise (yet unpublished data); (C) development and application of a tailored implementation strategy; (D) pilot testing and adjustment of the applied strategy, and (E) nationwide implementation.

The aim of the present pilot implementation study was to perform steps (C) and (D) and evaluate the adherence to recommended exercise behaviour on the levels of patients with axSpA, the fidelity to the new concept on the level of PTs, and the feasibility and satisfaction regarding the new concept, on the levels of patients, PTs and organisation. An additional objective was to investigate the effectiveness of PA recommendations at the level of patients. Furthermore, the long-term implementation fidelity was evaluated to gain lessons learned and inform the future nationwide implementation (step E).

## Methods

The Standards for Reporting Implementation Studies (STaRI) checklist [22] were followed to report theimplementation steps (Additional file 1), as well as the recommendations for reporting implementation strategies [23]. The study was registered (SNCTP000002880) and approved by the ethical board of Canton Zurich (BASEC-Nr. 2018-00145). PTs and patients gave written informed consent.

### Study design

The pilot implementation study had a hybrid type 3 design [24]. It was executed from June (T0) to November 2018 (T1), with evaluations at T1 and at T2 (T2 after 1 year). An implementation strategy, nested implementation activities and evaluation procedures for each of the relevant target groups were developed, i.e. patients with axSpA, PTs, and SVMB management. For details refer to Table 1 and “Implementation strategy” section below. The outcomes and assessments used to evaluate the implementation strategy and the effectiveness of the PA recommendations on the level of patients with axSpA, are described in Table 2 and below in “Evaluations” section.

**Table 1:** Elements of implementation strategy and implementation intervention activities according to proctor [23]

Element (s) of EG concept	Activity	Domain						
		Actors	Actions	Targets of change	Temporality (frequency)	Dose	Implementation outcomes affects; assessment	Justification
*Physiotherapists*								
(a)	Information	Study staff, self-study	Learn/recall characteristics of effectively dosed PA and exercise, purpose and methods of PA and exercise in line with actual guidelines/EULAR recommendations	Knowledge and awareness	Workshop before implementation in EGs	2 h	Knowledge, feeling of confidence in dealing with topic and promoting exercises; interview	Consciousness raising, self-reevaluation (both transtheoretical model)
(a)	Education	PRISM expert	Aim, methodology and practical application of PRISM [27] in 1:1 counselling situation; communication skills to motivate and support groups, manage difficult situations, and encourage participants to perform (more) exercise	Skills	Workshop before implementation in EGs	8 h	Use of and adherence to the counselling elements (PRISM, MI); diary	Guided practice (social cognitive theory)
(a)	Education	Study staff, self-study	Learned/recalled how to elaborate a progressive exercise plan	Skills	Workshop before implementation in EGs	1 h	Knowledge and development of exercise plans; diary	Guided practice (social cognitive theory)
(a)	Support	PT peer	PTs know each other and ask each other for support, exchange experiences	Peer-factor	n.d	n.d	Feeling supported; interview	Enhance network linkages (theory of social networks)
(c)	Supervision	Study staff	A "help desk" is provided by a study staff member to support in current issues. Regular refresher/supervision events with a PRISM expert are provided	Motivation to ensure quality of intervention	As needed	As needed	Beeing motivated; interview	Feedback (goal-setting theory, social cognitive theory)

Element(s) of EG concept	Strategy	Domain						
		Actors	Actions	Targets of change	Temporality (frequency)	Dose	Implementation outcomes affects; assessment	Justification
*Patients with axSpA*								
(b); (e)	Information	PTs, SVMB	PTs distribute handouts to patients and explain the four exercise dimensions and how to exercise appropriately. Questions are discussed in the EGs SVMB supports the knowledge transfer by proving information on website and SVMB journals	Knowledge and awareness of PAR	Weekly (PT) As needed (website) 4×/year (SVMB journal)	Ca. 5 min. As needed Ca.30 min. reading time	Awareness of how to perform effective exercises for each exercise dimension; exercise diary^a^	Consciousness -raising, self-reevaluation (both Transtheoretical model)
(b); (e)	Support	Peer patients	Suitable peers, who successfully perform regular exercise, act as role models to support peers from their EG	Empower peer-factor	n.d	n.d	Feeling supported; survey	Modelling (social cognitive theory)
(c); (d)	Individualisation	PTs	Assessments help to identify active and non-active participants and which of the exercise dimensions have the potential for improvement. An individual goal and action plan is developed during the 1:1 sessions in a shared decision manner	Setting goals, action planning, evaluation goal attainment	Bi-annual assessments Quarterly counselling sessions	45 min. 30 min	Performance in assessments and (frequency and dose) training in each exercise dimension; exercise diary^a^	Individualisation (Transtheoretical model) Tailoring (Transtheoretical model) Planning coping responses (Relapse Prevention Theory) Goal Setting (Goal Setting Theory) Set graded tasks (Social Cognitive Theory)
(c)	Individual exercise counselling	PTs	PTs and each patient have five 1:1 exercise counselling sessions, to define exercise goals, identify and deal with barriers and utilisation of facilitators, supporting self-efficacy, relapse prevention Furthermore, regular counselling sessions within the whole group are performed	Motivation and coping, self-efficacy	5 sessions within 18 months (3 sessions within first 6 months/pilot phase, 2 booster sessions)	30 min	Goal and action plan; exercise diary, activity tracker Changes according to PRISM in the patients perceived burden of disease and importance of PA; PRISM	Feedback (goal-setting theory, social cognitive theory) Reinforcement (social cognitive theory) Goal setting (goal setting theory) Set graded tasks (social cognitive theory)
(e)	Self-monitoring	Patients	Patients keep a diary about their exercise-activities, polar watches are provided for self-monitoring of the heart rate (not for data analysis)	Organisation in daily life/routine	Daily reporting	Ca. 2–5 min	Exercise is part of the daily routine; exercise diary, activity tracker	Implementation intensions (theories of goal directed behaviour) Feedback (goal-setting theory, social cognitive theory) Reinforcement (social cognitive theory)

Strategy	Domain						
	Actors	Actions	Targets of change	Temporality (frequency)	Dose	Implementation outcomes affects; assessment	Justification
*Organisation*							
Regular information exchange and discussion	Study staff	Study staff explains elements of the EG concept, which are discussed with SVMB management	Awareness, decision to change routine care	Every 3 months	30 min	Accept and implement new EG concept as usual care; vote of SVMB management	Sense-making (organizational development theory)
Acquisition of third-party funds Support negotiations with insurers	CEO, SVMB project leader	Responsible members of SVMB management establish personal and financial resources to continue all elements of the EG concept after the pilot phase	Embedding of new care in organi-sation	n.d	n.d	Establish finances and structures to maintain concept; staff positions, working hours/week	Technical assistance (organizational development theory)
Integration of SVMB-stakeholder in adaptation process	CEO, SVMB project leader	Study staff and SVMB management were in continuous exchange to ensure the feasibility of the new EG concept and satisfaction with its integration into routine care	Acceptance EG concept	Every 3 months	30 min	Feasibility and satisfaction; continous contact	Increasing stakeholder influences (stakeholder theory)

*n.d.* not defined**,** *SVMB* ankylosing spondylitis association of Switzerland, *PT* physiotherapist, *EG* exercise group, *PRISM* pictorial respresentation of illness and self measure

^a^ Paper or free app «Trainungstagebuch» by Johannes Tscholl

**Table 2:** Evaluation of the implementation strategy and the effectiveness of the recommended PA behaviour at the level of patients with axSpA

Evaluation of.	Patients with axSpA			Physiotherapists		Organisation	
	Outcome		Assessment (time of measure)	Outcome	Assessment (time of measure)	Outcome	Assessment (time of measure)
Implementation strategy	Primary: *Adherence* to recommended exercise behaviour		Exercise diary (continous reporting)	*Fidelity* to new EG concept	Diary (continuous reporting)	*Integration* within organisation	Quality concept (T1)
	Secondary: feasibility and satisfaction	Feasibility	Survey (T1)	Feasibility	Interview (T1)	Finances	Funding, status of negotiations (T1, T2)
		Burden of disease, importance of PA	PRISM (every counselling-session)			Personel resources	Working hours/week (T1, T2)
		Satisfaction (feeling supported)	Survey (T1)	Satisfaction	Interview (T1)	Satisfaction	Interview of CEO and staff (T1)
Effectiveness of interventions	Tertiary: effectiveness of recommended PA behaviour	Physical fitness in all exercise dimensions	Chester step test [61, 62]				
			Swiss olympic core test battery [63] adapted for patients with axSpA [64]				
			Single leg stance [65]				
			Bath ankylosing spondylitis mobility index/BASMI [66]				
		General physical activity	International physical activity questionnaire/IPAQ [67]				
		Disease activity	Bath ankylosing spondylitis disease activity Index/BASDAI [66]				
			Bath ankylosing spondylitis global score/BAS-G [68]				
		Health status	Assessment of spondyloarthritis international society/ASAS-HI [69]				
		Disease related quality of life	Euro-Quol Questionnaire [70]				

Time of effectiveness measures was T0 (baseline), T1 (after 6 months), T2 (after 12 months)

The tertiary outcomes are the regular fitness assessments in the exercise groups, which were used to evaluate the effectiveness of interventions. Assessments are described in Additional file 2

*PRISM* pictorial respresentation of illness and self measure, *PA* physical activity, *CEO* chief executive officer

#### Implementation strategy

##### Targeting patients and PTs: the new exercise group concept "moveSVMB"

A new EG concept encompassing the following key elements was introduced:

- *Training of supervising PTs*
  The supervising PTs attend a mandatory 2 days of training to learn how to apply the new concept within their exercise groups. The training consists of: gaining knowledge and awarenessof the general and EULAR PA recommendations and improving exercise counselling and motivational support skills. The monitoring of quality is performed by diary and peer-involvement through regular exchange betweenthe PTs and study staff. Besides communication and psychological theory, PTs learn the practical use of the instrument PRISM (Pictorial Representation of Illness and Self Measure, described in Additional file 2) [25] and, behavioural change techniques, such as goal setting and strengthening self-efficacy using role plays. Furthermore, study staff and PTs discuss thestudy processes. During the pilot intervention a refresher event and regular support is provided, e.g. "help desk" for current issues.
- *Correctly dosed exercises in all fitness dimensions*
  PTs instruct and perform exercises in all fitness dimensions during the exercise group sessions. According to the current knowledge of exercise guidelines, the dosage of exercises follows the FITT (Frequency, Intensity, Type, Time) principles [8, 13]. Patients are additionally informed about the importance and elements of adequate exercise through information provided by the SVMB (website, SVMB journal). Both activities enable patients to perform exercises alone.
- *Individual and group exercise counselling*
  PTs are asked to perform three individual exercise counselling sessions with each patient and at least three group interventions to improve patients PA behaviour.
  An individual counselling session lasts about 30 min. PTs are encouraged to use an empathic, supportive, yet directive counselling style, according to the principles of Motivational Interviewing [26]. PTs are asked to start each individual session by applying the PRISM, which is a visual-tactile instrument that fosters communication, collaboration and mutual confidence [25, 27]. PRISM can also be used to get to know a person better, by defining dimensions relevant to his/her life and, thus, enhancing intrinsic motivation and self-management activities [28]. After using PRISM and, based on the assessments of the patient’s fitness and his/her personal needs, the PT discusses with the patient how he/she can integrate more exercise into his/her everyday life. The fitness assessments help to identify the exercise dimension(s) with potential for improvement. In this regard, counselling may focus on raising consciousness, reinforcing the patients' PA behaviour, avoidance of relapse, or maintenance strategies. For example, some patients may need support in increasing the active time spent on light activities and reducing sedentary behaviour, while others may need a structured exercise plan to participate in a half-marathon. With each patient an individual goal and action plan is developed in a shared decision process.
  Group counselling interventions may vary in time (5–45 min) and can be designed differently, according to the needs and interests of the group. Examples are, (a) theoretical and practical training on heart rate self-monitoring during aerobic exercise, and (b) group discussion on how to cope with barriers.
- *Fitness assessments*
  During a regular exercise group session, a team of one senior (researcher) and several junior assessors (PT students) performs the fitness assessments with each participant, described as tertiary outcomes in Table 2 and Additional file 2. After the physical assessments, each patient is instructed to fill out six questionnaires at home. Furthermore, an exercise diary is explained and highly recommended, enabling feedback and reporting. Within 1 week, the individual assessment results are sent to the supervising PT for exercise counselling.
- *Individual, unsupervised exercise training*

To fulfil the recommended amount of PA, one supervised exercise group a week is insufficient. The elements mentioned above should enable group members to engage in additional, unsupervised exercise. Based on the fitness assessments and personal preferences, the patient and PT agree on a training goal in one, or several, of the exercise dimensions and create an action plan. During the following counselling session, progress is evaluated and the goal reviewed.

##### Targeting the SVMB

Regular meetings are held between SVMB management and study staff to ensure that the implementation activities align with the structures and processes of the SVMB. In addition, to increase the acceptance and feasibility of "moveSVMB", a significant effort is put into establishing a long-term financial strategy and personnel resources.

#### Evaluations

##### Evaluation at T1

The primary outcome for the evaluation at the patient level was adherence to the recommended exercise behaviour, assessed by the number of reported training sessions per exercise dimension in the electronic exercise diary for over 6 months. The secondary outcomes at T1 were feasibility and satisfaction with the new exercise group concept, rated on a 0–10 numeric rating scale (0 = not satisfied, 10 = very satisfied; if > 50% of patients rate > 5, the concept was evaluated as “satisfying”) and changes in the perceived burden of disease or importance of PA, measured with PRISM. The tertiary outcome to evaluate the effectiveness of the individualised PA recommendations was the measured changes in the fitness levels of patients (T0–T1), assessed by fitness parameters (Table 2).

The primary outcome for the evaluation atPT level was fidelity to the program design concept, assessed using a diary. Fidelity was rated as sufficient when 75% of PTs fulfilled the program. The secondary outcomes were treatment quality, feasibility and satisfaction, evaluated through semi-structured interviews.

The evaluation at the SVMB level focused on acceptance of the new exercise group concept and its establishment within organisational structures. In addition, regular progress meetings between study staff researchers and SVMB management were used to appraise the feasibility and effectiveness of the implementation interventions.

##### Evaluation at T2

Maintenance was monitored at T2 (1 year after T1) by assessing the number of patients and supervising PTs still adhering to the new group concept.

### Adaptations of "moveSVMB"

After 6 months, the findings of evaluations on all three levels were considered while deciding on adaptations of the program concept of "moveSVMB". Both SVMB management and study staff agreed on findings that negatively impacted the feasibility. Accordingly, they developed adaptations needed for a successful nationwide implementation of the new exercise group concept.

### Study procedures

The four pilot exercise groups, comprising the supervising PTs and 5–12 patients with axSpA, from the German-speaking region of Switzerland were asked by the SVMB management to volunteer. Criteria were a location close to the study center and the motivation and availability of PTs and group members. The partipicant PTs and patients were informed face-to-face about the new group concept "moveSVMB" well in advance. Group members decliningto be measured or not providing written informed consent were offered the usual exercise group intervention.

Eligibility criteria for patients with axSpA were ≥ 18 years old, a member of a SVMB exercise group, and provision of written informed consent. All patients were screened for cardiovascular risk [29]. Patients identified as at increased risk required confirmation from a PT or medical doctor that a higher level of exercise was not contraindicated. Criteria for exclusion were medically relevant contradictions to exercise [8]. Eligibility criteria for PTs were being a superviser PT of an existing SVMB group, willingness to follow the implementation protocol of the new exercise group concept (including a 2 day educational workshop), and provision of written informed consent.

At the beginning of the pilot study, some organisational requirements were defined. A cooperation with the BSc in physiotherapy programme at the ZHAW was established to recruit second year PT students to perform the fitness assessments in exercise groups under the supervision of a senior researcher. Additionally, third-party funding was secured prior to the start of negotiations with insurers concerning reimbursement. Throughout the pilot phase, the SVMB provided personnel (project manager, c.a. 17 h/week (40%), additional IT support), and was prepared to increase resources after evaluation of the pilot phase.

### Statistics

Demographic, disease-specific, self-reported (diary or patient-reported outcome measures PROMS) and assessment data were presented as mean and standard deviations, or median and associated range for continuous data or as frequencies (percentages) for categorical variables. During the fitness tests, the maximal time of one leg stance was limited to 60 s, this being the length of time indicating that the individual has no balance problem. For each exercise dimension, a linear mixed model with random intercept was fitted to the data with the within-subject variables of time and measurement condition and the between-subject variables of age, disease duration, disease activity (Bath Ankylosing Spondylitis Mobility Index BASMI), and PA level (metabolic equivalent of tasks METs) as explanatory variables. PRISM data (in cm) were correlated with the Bath Ankylosing Spondylitis Disease Activity Index (BASDAI) and weekly METs using spearman rank correlation. Paired t-tests and Wilcoxon tests, with corresponding parametric and non-parametric effect sizes (ES), were computed to analyse within-group changes. All statistical analyses were performed using the R statistical software, R version 3.5.2 (R Foundation for Statistical Computing, Vienna, Austria).

## Results

### Demographic and clinical data of exercise group members

Of the 43 patient members of the four pilot groups, 30 (70%) signed informed consent and participated in the baseline assessments. Reasons for non-participation were reported by ten members of the groups: timing of assessments due to holidays (3), lack of time (2), age or disease activity (2), no interest (2), no axSpA (1). The characteristics of the four pilot groups are described in Table 3.

**Table 3:** Characteristics of axSpA patients and supervising PTs at baseline

Characteristics	Total	Group 1	Group 2	Group 3	Group 4
*Patients with axSpA*					
Total number per group (n)	43	8	11	8	16
Number participating in “moveSVMB” (n)	30	3	6	7	14
Gender, women, n (%)	10 (33.3)	1 (33.3)	2 (33.3)	2 (28.5)	5 (35.7)
Years of age (median, range)	57.5 (37–75)	68 (37–72)	58 (51–71)	57 (45–68)	56.5 (49–75)
Disease duration, years (median, range)	30 (12–60)	49 (23–51)	33 (20–46)	30 (18–35)	30 (12–60)
ASAS-HI (median, range)	2.1 (0–11)	1 (0–3)	2.1 (0–6)	2 (0–8)	3 (0–11)
Physical activity level (IPAQ), total MET per week (median, range)	3919 (1520–14,958)	3916 (2526–6933)	5820 (2298–10,260)	2697 (1600–10,638)	2853 (1520–14,958)
*Supervising PTs (n* = *4)*					
PT group leader, gender (female/male)	–	f	f	f	m
PT group leader, age, years (median, range)	48.2 (34–54)	52	45	34	54
PT group leader since, years (median, range)	12 (2–23)	23	8	2	16

*n* number, *ASAS-HI* assessment of spondyloarthritis international society health index, *PT* physiotherapists, *IPAQ* international physical activity questionnaire

During the 6-month pilot phase, two patients dropped out due to specified reasons: "I do not need exercise counselling", and "it is too much pressure for me". In addition, two patients did not participate in the assessments after 6 months due to an influenza.

### Results at the level of patients with axSpA

#### Adherence to recommended exercise behaviour

Thirteen patients (43%) submitted an exercise diary each week to assess adherence to PA recommendations. None of these thirteen people fulfilled the PA recommendations in all four exercise dimensions. Four patients fulfilled the recommended dose of weekly cardiovascular exercises, one the recommendation of one additional exercise dimension, and two patients the recommendations of two additional exercise dimensions.

#### Feasibility and satisfaction

The survey's response rate was 90% (27/30). Since two were returned empty, 25 were used for analysis. Of these, 15 (62%) patients were satisfied with the concept "moveSVMB" (mean satisfaction 6.7 ± 2.2) and 20 (80%) with the exercise counselling (mean satisfaction 7.7 ± 2.9). Patients rated and commented the use of PRISM contrary (11 “not useful” vs. 12 “useful”); appreciating ("I understand the relation between axSpA and my life" (*ID15*)) or criticising ("this is coffee cup reading to me" (*ID05*)).

Twenty-two (88%) patients defined a training goal, 17 (68%) developed an action plan and 15 (60%) achieved their training goal. Sixteen (64%) patients used the exercise diary, five used app-based reporting. Reasons for non reporting were: the daily effort, technical problems with the app, and problems with allocating activities to exercise dimensions correctly (e.g. is the SVMB exercise group strength or neuromotor exercising?). Personal adaptations were made to individual exercise goals (e.g. in terms of frequency or intensity of training) and to individual exercise diaries (e.g. notes in the personal agenda instead of the proposed sheets). A minority (n = 7, 28%) reported using a technical device (such as a heart rate tracker) for cardiovascular exercise. Seventeen (68%) were satisfied with the fitness assessment (mean 7.5 ± 1.8). Patients did not use the project name "moveSVMB" but suggested names such as "Bechterew-Fit" or "Bechterew-Gymnastics".

#### Changes according to PRISM

Neither the perceived burden of disease (distance to self at T0: 13.7 ± 7.2 cm; at T1: 12.1 ± 6.2 cm; t = 0.386, *p* = 0.703) nor the importance of PA (distance to self at, T0: 7.3 ± 5.3 cm; at T1: 7.0 ± 4.5 cm); t = 0.246, *p* = 0.808) changed over 6 months. The perceived importance of PA correlated with the International Physical Activity Questionnaire (IPAQ) measured METs at T1 (r = 0.572, *p* = 0.00), no correlation between perceived burden of disease and METs was found.

#### Effectiveness of individualised PA recommendations: fitness status

Table 4 reports the results of the fitness assessments at baseline (T0) and after 6 months (T1). Patients' cardiorespiratory fitness [ES 1.22 (95%CI 0.59, 1.90), large ES and significant] and core strength ventral muscle chain [ES 0.62 (95%CI 0.19, 1.06), medium ES and significant] improved over the 6 months. Core strength lateral and dorsal muscle chain, balance and flexibility did not change significantly over 6 months.

**Table 4:** Results of fitness assessments of the whole patient sample at T0 (baseline) and T1 (after 6 months)

*Fitness assessment*	T0	T1		ES
n missing values	(n = 30)	(n = 30)	Change (n = 30)	(95% confidence interval)
mean (SD)				
median (IQR 25/75)				
*Cardiorespiratory fitness, VO2max*				*1.22 (0.59, 1.90)
n missing values	4	11	13	
mean (SD)	35.2 (6.5)	41.6 (6.5)	7.9 (6.5)	
median (IQR 25/75)	34.5 (32.6/39.7)	39.9 (38.2–46.0)		
*Core strength, ventral, sec*				*0.62 (0.19, 1.06)
n missing values	1	5	5	
mean (SD)	78.8 (51.9)	107.5 (65.4)	23.8 (38.6)	
median (IQR 25/75)	69.0 (40.0–100.0)	90.0 (64.0–149.0)		
*Core strength, lateral, sec*				0.17 (0.01, 0.55)
n missing values	1	5	5	
mean (SD)	49.0 (31.5)	57.9 (31.1)	7.6 (30.2)	
median (IQR 25/75)	39.0 (29.0–66.0)	51.0 (38.0–82.0)		
*Core strength, dorsal, sec*				0.30 (0.02, 0.65)
n missing values	1	7	7	
mean (SD)	58.5 (50.1)	71.7 (56.6)	9.7 (35.5)	
median (IQR 25/75)	35.0 (24.0–80.0)	59.0 (32.5–85.5)		
*One leg stance, open eyes, mean of 3 reps, sec*				0.10 (0.00, 0.48)
n missing values	0	4	4	
mean (SD)	43.1(21.1)	46.6 (19.6)	3.7 (14.0)	
median (IQR 25/75)	60.0 (24.5–60.0)	60.0 (34.0–60.0)		
*One leg stance, closed eyes, mean of 3 reps, sec*				0.30 (0.02, 0.63)
n missing values	0	4	4	
mean (SD)	7.7 (5.8)	9.5 (7.8)	1.7 (5.5)	
median (IQR 25/75)	5.5 (5.0–15.5)	7.9 (3.0–14.9)		
*BASMI, Score*				*− 0.28 (− 0.68, 0.12)
n missing values	0	4	4	
mean (SD)	3.1 (2.3)	2.9 (2.0)	− 0.2 (0.9)	
median (IQR 25/75)	2.4 (1.2–4.5)	2.3 (1.2–4.7)		
*IPAQ, MET per week*				0.15 (0.00, 0.58)
n missing values	1	9	10	
mean (SD)	5013 (3479)	4101 (4262)	− 199.8 (2712.2)	
median (IQR 25/75)	3916 (1520–14,958)	3150 (1470–4502)		
*BASDAI*				*0.06 (− 0.39, 0.51)
n missing values	1	9	10	
mean (SD)	3.0 (1.7)	3.0 (2.2)	0.0 (1.1)	
median (IQR 25/75)	3.0 (1.8–4.1)	2.2 (1.3–4.4)		
*BAS-G*				0.02 (0.00, 0.49)
n missing values	1	9	10	
mean (SD)	2.9 (1.8)	2.6 (1.8)	− 0.2 (1.6)	
median (IQR 25/75)	2.4 (1.4–4.5)	2.2 (1.1–3.7)		
*ASAS-HI*				0.12 (0.00, 0.54)
n missing values	1	9	10	
mean (SD)	3.0 (2.6)	2.5 (0.7)	− 0.8 (3.3)	
median (IQR 25/75)	2.1 (1.0–4.0)	3.0 (2.0–3.0)		

Effect sizes (ES) were computed from Wilcoxon tests and paired t-tests, using r for non-parametric and cohen’s d for parametric* analysis, respectively. Interpretation of ES: r: 0.1–0.3 (small), 0.3–0.5 (moderate) and > 0.5 (large). Cohen’s d: 0.2 (small), 0.5 (medium), 0.8 (large)

*IQR* interquartilrange, *sec* seconds, *SD* standard deviation, *BASMI* bath ankylosing spondylitis mobility index, *reps* repetitions, *IPAQ* international physical activity questionnaire, *BASDAI* bath ankylosing spondylitis disease activity index, *BAS-G* bath ankylosing spondylitis patient global score, *ASAS-HI* assessment of spondyloarthritis international society health index

The linear mixed model analysis showed that disease activity was negatively (slope = − 1.49, *p* = 0.01) associated with cardiorespiratory fitness and the PA level (MET) positively (slope = 3.28, *p* = 0.01). The PA level was also positively associated with core muscle strength (slope = 8.62, *p* = 0.05). Age was positively associated with flexibility (slope = 0.10, *p* = 0.04), but negatively associated with balance time (slope = − 0.84 (*p* = 0.00).

### Results at the PT level

#### Fidelity to the new exercise group concept

Three of the four PTs (75%) performed and reported on three group counselling sessions within the 6 months, with a mean duration of 18 (± 13) minutes. Due to a misunderstanding, one PT did no group counselling sessions. The counselling sessions consisted of knowledge transfer (e.g. reading and discussing PA recommendations) and skills training (e.g. training with a heat rate monitor). The number of patients participating in the group counselling interventions varied between four and twelve per session and group.

All four PTs performed and reported three individual exercise counselling sessions with 27 of 30 patients (90%). Two patients dropped out after the first session andne patient participated in only two sessions before leaving the group for personal reasons. Of the 27 patients receiving the three counselling sessions, 11 (40%) received the three counselling sessions within 6 months, as required by the concept; 10 (37%) within 9 months, and six (22%) within 12 months. The delay was due to organisational factors in all cases.

The mean duration of an individual counselling session was 35 (± 7) minutes. During the first session, the PTs used the PRISM to take the patient’s history, explain the results of the fitness assessment and, through shared decision-making, to define one, or more, training goals. Subsequently, based on the patient’s preferences, an action plan was developed. During the second and third sessions, PTs used PRISM to evaluate, together with the patient, whether the goal(s) had been reached, or whether adaptations to the goals or planned actions were needed due to changes in fitness status or PA-behaviour.

The mean duration of PRISM usage was 9 (± 3) minutes during the first session, 10 (± 4) minutes during the second, and 7 (± 3) minutes during the third.

Using PRISM, PTs measured and reported on the burden of disease and importance of PA for 25 participants.

#### Feasibility and satisfaction

PTs perceived the PRISM communication and exercise counselling as very useful, since they previously had little knowledge of the history and exercise goals of their patients. Exercise counselling supported the possibility of personalized exercise. They emphasised that the frequency of the sessions must be flexibly planned, based on the patient’s individual training goal and motivation. The PRISM was perceived as a useful door opener, triggering emotions and information, if the patient’s attitude was open to the new communication tool. PTs reported that they initially felt unconfident when applying PRISM; this feeling was diminished during the pilot phase through supervision and practice. The PTs perceived varyingreactions from patients regarding the fitness assessments, some found that they increased motivation to exercise and others found them demotivating. During exercise counselling, PTs indicated that they found the fitness assessment data difficult to interprete because of the lack of background information (e.g. norm data) and that they rarely relied on them. The PTs felt, that the frequency of fitness assessments could be reduced from bi-annually to annually.

### Results at the organisational level

Satisfaction and commitment of the SVMB management was found to be high after the pilot phase. Consequently, the SVMB management decided to integrate the new exercise group concept into the organisation and implement it as the new usual care in all its SVMB exercise groups.

### Maintenance

After 1 year (T2), the same supervising PTs were responsible for their exercise groups and were actively involved in the nationwide implementation process. The four pilot exercise groups lost one member due to personal reasons, but gained two newmembers. Therefore, 29 patients actively participated in the programme, of whom 21 (72%) participated in the fitness assessments at T2.

### Adaptations to the implementation strategy

Some implementation activities were found to be not fully feasible in our setting. Minor adaptations were made to improve adherence to the recommended exercise behaviour (patients), concept fidelity (PTs), and feasibility ofnationwide implementation (SVMB). The barriers and adaptations are described in Table 5.

**Table 5:** Adaptations of Implementation Strategy to increase feasibility for nationwide implementation

Strategy	Target of change	Barriers	Adaptation
*Physiotherapists*			
Education	Skills	Two-days workshop was too expensive and too time-consuming. Future supervising PTs do not need information on study procedures	Reduction to 1-day workshop + supervision
*Patients with axSpA*			
Individualisation, Exercise counselling	Motivation and coping, self-efficacy	Due to organisational reasons, PTs had three exercise counselling sessions with only 40% of group members within 6 months	Number of exercise counselling sessions reduced to 1–2 per year
		Group setting was not asked for, but 1:1 setting was appreciated	No fixed, mandatory group discussions
Individualisation, Exercise counselling	Setting goals, action planning, evaluation goal attainment	PTs did not use the assessments to define exercise goals, as there were no norm data or previous data available	New project on norm data will be launched. The more measurements are carried out, the more comparisons exist and enable a better interpretation
		The exercise diary was approved as a useful tool. Paper version needs adaptations. Electronic version (“Trainingstagebuch” by Johannes Tscholl) prone to error	Paper version adapted (better explanations, examples)
			Development of electronic version is currently considered by SVMB IT
*Organisation*			
Integration of stakeholders	Acceptance of EG concept	62% satisfied with EG concept, 80% satisfied with exercise counselling, 68% satisfied with fitnessassessments. «MOVE SVMB» was not used by participants	«MOVE SVMB» changed to «BeFit» and a new logo was released

*PA* physical activity, *SVMB* the ankylosing spondylitis association of Switzerland, *PT* physiotherapist, *EG* exercise group, *PRISM* pictorial respresentation of illness and self measure

Exercise counselling and the fitness assessments were generally appreciated by the patients, but the frequencies of both was perceived as being too high. Accordingly, the fitness assessments were reduced from bi-annually to annually, and the individual exercise counselling sessions reduced from three to two sessions in the first 6 months. The first session was compulsory for all participants and the second dependent on individual needs and training goals. The group counselling sessions were perceived to be useful, but the definition of frequency and structure was removed. There is a great need for a simple exercise diary, covering all the exercise dimensions. An improvement was made to the paper diary through providing better explanations. This version will be used until a digital exercise diary becomes available.

The 2 day educational workshop for PTs was thought to be too time-consuming for clinicians. Furthermore, the information strategy of the SVMB had already increased awareness and knowledge ofthe new concept amongst thePT community. The training will therefore be reduced to 1 day and the workshop will focus on exercise counselling (including behavioural change techniques and PRISM).

Based on suggestions from patients, the name "moveSVMB" was changed by the SVMB to BeFit ("Bechterew Fitness") and a new logo was developed. Following the pilot phase, information about BeFit was released by the SVMB, with no involvement of the study staff. In addition, the financial and personnel resources were established to ensure a continuation of the implementation.

## Discussion

To our knowledge, this is the first study to translate and implement PA recommendations within a specific patient organisation into an exercise group concept for patients with axSpA and their supervising PTs. Findings will inform the nationwide implementation in Switzerland and similar projects in the Netherlands.

The findings of the pilot phase found the implementation strategy was acceptable and satisfactory to patients, PTs, and the SVMB. This resulted in SVMB management adopting the exercise group concept as its new usual care and implementing it in all its exercise groups. Successful implementation can only happen if the SVMB takes responsibility and initiates crucial changes at organisational level. Findings generally confirm the value of evidence-based exercise groups provided by a patient organisation to support active PA behaviour.

The patients' adherence to recommended exercise behaviour was assessed as insufficient. Reasons for non-adherence of patients may be two-fold: (1) The assessment of adherence to recommended exercise behaviour in patients with axSpA was difficult due to technical problems with the app and possible reporting bias; (2) the adherence was low in terms of performing insufficient amount of exercise according to recommended frequency and dose, as well as reporting exercise sessions. According to the reporting, only four patients fulfilled the recommended amount of aerobic exercise, three of them fulfilled one or two additional exercise dimensions. Factors like limited personal motivation may moderate the level of adherence to change current exercise behaviour or difficulty to achieve the agreed goals. However, it would have been useful to have learned more about the attitude of the patients towards changing their PA behaviour through asking them, prior to the first counselling session, about their satisfaction with their current PA level and whether they were motivated to increase their exercise behaviour. To show improved fitness in exercise dimensions, specific and adequately dosed training is needed. The implementation fidelity in PTs was found to be good, with their adherence to performance and the reporting of exercise counselling beeing sufficient. The level of adherence was affected by factors such as patient' responsiveness, quality of counselling, and organisational aspects (e.g. time constraints). However, only small organisational adaptations needed to be made by the PTs, according to the individual local group setting, such as to the scheduling and the setting of counselling sessions. The reduced number and more flexible planning of counselling sessions should enhance feasibility in the future and result in improved performance (during pilot study only 40% of patients had the counselling sessions within 6 months).

The four pilot PTs could be viewed as a special group since they were presumably "innovators", an active group keen on new ideas and innovations [30, 31]. The pilot PTs were immediately willing to adapt their usual care and confirmed the value of change. Grol and Wensing describe five phases in the process of change for health professionals: orientation, insight, acceptance, change, maintenance [19]. Other supervising PTs might be harder to persuade of the benefits of the new concepts, since old routines need to be first de-implemented to move from acceptance to change and maintenance. Therefore, clear leadership by the SVMB management and the pilot PTs, as role models, are essential for the further implementation among supervising PTs.

Patients reporting could be improved in future though technical applications, which might be more appropriate to the collection of objective data than a paper diary [32]. These could also be used as a promotion strategy (e.g. feedback, reinforcement) [33, 34]. However, the assessment of PA and reporting on all fitness dimensions simultaneously in the same tool is a big challenge, and a perfect tool is not yet available [35, 36]. The adapted paper version of the diary must serve as a placeholder until an electronic solution is developed.

PT needs two critical competencies to work with an exercise group: detailed knowledge of the PA recommendations and communication skills. Even though PA promotion is a key concept of physical therapy [37], and both patients and PTs agree that advice and support of active PA behaviour is part of the therapist’s role [38, 39], there seems to be a PT educational gap. Data from the UK and Canada indicate that the majority of PTs neither have detailed knowledge of PA recommendations, nor meet the recommended PA level themselves [40, 41].

A preliminary analysis of counselling sessions has revealed that during the first counselling session one of the techniques most used by PTs to change behaviour [42] was ‘general intention building’. No specific goal setting technique was applied (unpublished data). PTs generally use only a few behavioural change techniques [43]. This finding underpins the need for continuous training of PTs in communication and behaviour change techniques. Hilberdink and colleagues [44] have mapped a toolkit to optimise exercise behaviour of people with axSpA: (1) Patients should be offered behavioural change guidance, including education, motivational interviewing, goal setting, action planning, monitoring and feedback. Patients need to be encouraged to exercise in a group setting. (2) PTs need to be educated to tailor and instruct an exercise program and apply behaviour change techniques. Some first effort is made to downscale the lack of evidence-based education and training for health professionals working with rheumatic conditions [45]. Others [9, 44, 46] emphasise that interventions are needed for patients with axSpA including behaviour change techniques, that aim to deliver intrinsic motivation to overcome barriers to exercise [47–49] and to sustain an active lifestyle.

In this project, it took several years to determine the appropriate implementation process, following the cyclical approach of Grol and Wensing's implementation of change model [19]. During this development period Koorts and colleagues [47] published the PRACTIS guide, which supports the planning and scaling-up of physical activity implementation projects. PRACTIS focuses more specifically on PA promotion research translation and gives detailed “"how to” support. Both approaches have a common ground and are appropriate theoretical approaches to describe an implementation process and to explain what influences implementation outcomes and the implementation success. One strength of this pilot project is that an implementation scientist was involved from the start, following the "innovative collaborative model" [48]. Further strengths are the in-depth contextual analysis that has been undertaken [20, 21] and the stakeholder involvement from the beginning.

The group setting for exercise therapy is considered equally effective as individual therapy, but with lower healthcare cost [49]. Furthermore, group therapy is attributed a potentially motivating factor for physical activity behaviour change [50]. Despite this knowledge, however, the social rules or "group dynamics" [51] within the pilot groups and their impact on individual motivation or PA adherence was not investigated in this study. Social learning [52] within group activities (counselling, and assessment) appears to be an important subject and should be investigated further in future studies.

The sample size of the four pilot groups with 30 patients was small. However, since effectiveness aspects were not the main focus, no prior sample size calculation or bias-prevention, such as blinding, were necessary. According to literature, men and women are equally affected by axSpA [53]. However, in the pilot groups there were more men than women participated (2:1 ratio). A further limiting factor is the use of self-reported outcomes and not of validated assessments to measure the implementation success. However, this approach was pragmatic and feasible. Future research projects should try to overcome these limitations. The exercise group concept could be a valuable strategy in enhancing patients' daily exercise routines. However, fulfilling the PA recommendations in all exercise dimensions is a very high goal. Continuous monitoring of the exercise group concept is needed to follow-up on patients' exercise behaviour and further improve the strategies of how to fulfil the PA recommendations.

Many patients prefer individual therapy. For example, 32% of the Swiss axSpA patients prefer individual, mainly inactive therapy [20]. Dutch patients participating in axSpA exercise groups, however, were positive about subsequently established additional home exercises, annual assessments and training using heart-rate monitors [54]. To reach patients who prefer an individual setting, or the inactive therapy approach, a patient education and PA promotion concept supporting the understanding of exercise as medicine needs to be developed.

Further research is also needed to investigate the effects on patients with axSpA of strength or neuromotor exercises alone, not in combination with cardio-respiratory exercises, since no such studies have been conducted as yet [11]. It has also previously been stated that flexibility exercises are beneficial to spinal flexibility [2]. However, no studies on flexibility exercises alone were found althoughexercises combined with muscle strength or aerobic exercise did not affect flexibility [11]. Recent studies have confirmed that intensive cardio-respiratory exercise is effective, safe and feasible in patients with axSpA [55]. No detrimental effects were shown, rather a beneficial impact on disease activity and risk of cardiovascular diseases was approved [9, 56, 57]. Similar strong evidence is lacking for the other fitness dimensions. A recently published review confirmed that exercise intervention studies in people with inflammatory arthritis are not adequately reported, neither was the exercise dose underpinned with evidence [58]. Furthermore, better knowledge of adherence patterns, e.g. coping mechanism related to PA, or patient profiles matching group versus individual exercise modes, is needed [59].

During a national implementation, continuous monitoring will be needed to avoid ineffective elements of the exercise group concept [60]. The balance between fidelity (degree of adherence) to and adaptation of the implementation strategy and interventions (the degree to which users modify the strategy and interventions to suit local needs) should be monitored and evaluated. A threshold for patient adherence should be defined for better evaluation of an implementation success. Furthermore, economic analysis on the levels of patient, provider, organisation and system should be performed to evaluate the concept’s efficacy from an economical point of view.

## Conclusions

This manuscript describes an implementation strategy to translate PA recommendations for patients with axSpA into a new exercise group concept for clinical practice and to embed then into the routine process (usual care) of a patient organisation. The implementation strategy was successfully evaluated in a pilot study including key stakeholders: patients, PTs, and patient organisation. The implementation project addressed a need at organisational level through delivering evidence-based therapy to patients and enhancing the competencies of the supervising PTs. Based on the results of the pilot study, technical problems, which hampered a reliable evaluation of the adherence rate of patients, and some other adaptations were necessary to increase the feasibility for a nationwide implementation. Further work is required in order to improve the quality of the assessments, the exercise counselling, and the individual exercising. The successful pilot strategy and necessary adaptations are explained in detail to make the knowledge available to future translational research projects in the field of RMDs. The upcoming nationwide implementation process needs to be monitored so that implementation problems or changes in the needs of patients with axSpA, PTs, or SVMB can be addressed early.

## Supplementary Information

**Additional file 1.** Standards for Reporting Implementation Studies: the StaRI checklist for completion.

**Additional file 2.** Description of assessments (PRISM, fitness assessments, interview with PTs).

## Data Availability

Please contact authors for data requests.
