# Supplementary material for: Lessons learned from a pilot implementation of physical activity recommendations in axial spondyloarthritis exercise group therapy
Source: BMC Rheumatol. 2022 Jan 17;6:12. doi: 10.1186/s41927-021-00233-z (PMC8762948; doi:10.1186/s41927-021-00233-z)
Supplement: Supplementary file 2 — Additional file 2. Description of assessments (PRISM, fitness assessments, interview with PTs). [file 41927_2021_233_MOESM2_ESM.docx]

**Additional file 2: Description of Assessments**

**PRISM (Pictorial Representation of Illness and Self Measure)**

The PRISM is a visual-tactile instrument that fosters communication, collaboration and mutual confidence [Büchi 1999, Büchi 2002]. It comprises a white A4-sized metallic board with a yellow disk at the bottom right-hand corner. The person is asked to imagine that the board represents their life and the yellow disk represented their "Self". Then, a red disk representing axSpA and after, a blue disk representing PA, are handed to the person. They are asked to place the disks on the board where they best represent their importance in the person’s life. The distances between the Self and the red and blue disks, respectively, are measured in centimetres (cm) to quantify the perceived burden of disease (red disk) and the importance of PA (blue disk) in their lives.

**Chester Step Test (CST)**

The CST is a submaximal test for estimating aerobic capacity [Sykes 2004]. The subject steps up (on a step) and back down again in a metronome-controlled manner. The frequency is increased every two minutes. The test is terminated when 80% of the maximum heart rate, or the perceived exertion measured with the Ratings of Perceived Exertion Scale (RPES), reaches a value of 14 (on the scale from 1 to 20). The step height is variable and can be adjusted in relation to body size, which makes the test feasible for people of different ages and fitness. The CST is inexpensive and easy to standardise and is considered a valid and reliable assessment [Bennet 2016]. Aerobic capacity is estimated using maximum oxygen capacity (VO2max) [Sykes 2004, Buckley 2004]. In this study, the CST was measured using the Chester Aerobic Tests software version 0.9.11.277 (Cartwright Fitness Ltd., Chester, England).

**Core Strength Test Battery**

The isometric core strength endurance test battery was originally developed by the Swiss Olympic Medical Center for use with athletes [BASPO 2015], but has been validated for people with axSpA [Rausch 2020]. The test battery was used to measure the isometric strength endurance of the ventral, lateral, and dorsal core muscle chains (measured in seconds).

**Single leg stance (SLS)**

The SLS is easy, quick to perform and reliable for measuring balance. Normative data is available^35^. It is widely used in physiotherapy for measuring balance.

The SLS is a valid, reliable, and easy-to-perform assessment for static balance [Springer 2007]. It is measures how long (seconds) an individual can stand on one leg with either eyes open or closed.

**Bath Ankylosing Spondylitis Mobility Index (BASMI)**

The BASMI is an established assessment for measuring flexibility in people with axSpA. It is quick to administer, valid, reliable, reproducible, and sensitive to changes across the disease spectrum [Sieper 2009]. The BASMI scores range from 0 (no limitation of spinal flexibility) to 10 (severe limitation).

**Bath Ankylosing Spondylitis Disease Activity Index (BASDAI)**

The BASDAI was used to measure the AxSpA disease activity, The BASDAI is a valid and reliable self-reporting questionnaire [Sieper 2009], consisting of six items to determine pain in the peripheral joints and spine, fatigue, morning stiffness, and pain sensitivity to touch. The BADAI results in a mean score of between 0–6 points (0 = no disease activity, 6 = highest disease activity).

**Bath Ankylosing Spondylitis Global Score (BAS-G)**

The BAS-G reflects the effect of axSpA on the patient’s well-being during the previous week and previous six months [Jones 1996]. Patients rate the effect on a numeric rating scale (0= none, 10 =very severe). The higher the mean score, the higher is the effect of the disease on the patient’s well-being.

**Assessment of SpondyloArthritis international Society (ASAS-HI)**

The ASAS-HI was developed according to the International Classification of Functioning, disability and health (ICF) core set for axSpA. The questionnaire consists of 17 items and is used as an instrument to measure the function, disability and health of patients with Spondyloarthritis [Kiltz 2014].

**Euro-Quol Questionnaire (EQ-5D)**

The EQ-5D consists of five domains including mobility, self-care, usual activities, pain/discomfort, and anxiety/depression and a visual analogue scale asking individuals to indicate the perceived level of health [Graf 1998].

**International Physical Activity Questionnaire (IPAQ)**

The self-administered short version of the IPAQ was used [Hallal 2004, IPAQ group]. The IPAQ considers a 7-day recall period, quantifying the time of being moderately or vigorously physically active.

**Semi-structured Interview with PTs**

The interviews were conducted by telephone. The main questions were:

- In preparation for the project, you took part in a workshop. How did you experience it and were you sufficiently prepared to start the project?
- In the workshop, you were introduced to the PRISM. What experiences have you had with PRISM since then?
- Did your role as group physiotherapist change?
- How do you experience the counselling?
- Do you use/recommend technology (tracker, reminder function, heart rate monitor) to organize and conduct training? The study participants have been given a heart rate monitor to perform cardiovascular training. What is the feedback on this?
- Assessments are now performed regularly. How do you experience conducting the assessments?
- What experiences do you have of the SVMB?
- Which elements of the new concept (fitness tests, counselling, the PRISM as a communication tool) do you think is important/not important?

**References**

BASPO BfS. Manual Leistungsdiagnostik. 2015.

Bennett H, Parfitt G, Davison K, Eston R. Validity of Submaximal Step Tests to Estimate Maximal Oxygen Uptake in Healthy Adults. Sports Med. 2016;46(5):737-50

Buchi S, Buddeberg C, Klaghofer R, Russi EW, Brandli O, Schlosser C, et al. Preliminary validation of PRISM (Pictorial Representation of Illness and Self Measure) - a brief method to assess suffering. Psychother Psychosom. 2002;71(6):333-41

Buchi S, Sensky T. PRISM: Pictorial Representation of Illness and Self Measure. A brief nonverbal measure of illness impact and therapeutic aid in psychosomatic medicine. Psychosomatics. 1999;40(4):314-20

Buckley JP. Reliability and validity of measures taken during the Chester step test to predict aerobic power and to prescribe aerobic exercise. British Journal of Sports Medicine 2004; 38: 197–205

Graf J, Claes D, Greiner W. The German version of the EuroQol Questionnaire. Z Gesundh Wiss. 1998;6:3-20.

Hallal PC, Victora CG. Reliability and validity of the International Physical Activity Questionnaire

(IPAQ). Medicine and science in sports and exercise. 2004;36(3):556.

IPAQ group, <https://sites.google.com/site/theipaq/>

Jones SD, Steiner A, Garrett SL, Calin A. The Bath Ankylosing Spondylitis Patient Global Score (BAS-G). Br J Rheumatol. 1996;35(1):66-71.

Kiltz U, van der Heijde D, Boonen A, Braun J. The ASAS Health Index (ASAS HI) - a new tool to assess the health status of patients with spondyloarthritis. Clin Exp Rheumatol. 2014;32(5 Suppl 85):S-105-8.

Rausch AK, Baltisberger P, Meichtry A, Topalidis B, Ciurea A, Vliet Vlieland TPM, et al. Reliability of an adapted core strength endurance test battery in individuals with axial spondylarthritis. Clinical rheumatology. 2020.

Sieper J, Rudwaleit M, Baraliakos X, Brandt J, Braun J, Burgos-Vargas R, et al. The Assessment of SpondyloArthritis international Society (ASAS) handbook: a guide to assess spondyloarthritis. Ann Rheum Dis. 2009;68 Suppl 2:ii1-44.

Springer BA, Marin R, Cyhan T, Roberts H, Gill NW. Normative Values for the Unipedal Stance Test with Eyes Open and Closed: Journal of Geriatric Physical Therapy 2007; 30: 8–15

Sykes S, Roberts A. The Chester step test—a simple yet effective tool for the prediction of aerobic capacity. Physiotherapy 2004; 4: 183–188
